# Disparities in Diabetes Prevalence Among Native Hawaiians/Other Pacific Islanders and Asians in Hawai‘i

**DOI:** 10.5888/pcd16.180187

**Published:** 2019-02-21

**Authors:** Olivia Uchima, Yan Yan Wu, Colette Browne, Kathryn L. Braun

**Affiliations:** 1 Office of Public Health Studies, University of Hawai‘i, Honolulu, Hawai‘i; 2 Ha Kūpuna National Resource Center for Native Hawaiian Elders, Myron B. Thompson School of Social Work, University of Hawai‘i, Honolulu, Hawai‘i

## Abstract

**Introduction:**

The prevalence of diabetes varies widely among racial/ethnic groups in Hawai‘i. How prevalence varies by age for Asian subgroups and Native Hawaiian/Other Pacific Islanders (NHOPIs) is understudied. We examined diabetes prevalence by age and race/ethnicity and assessed how socioeconomic status and lifestyle behaviors affected prevalence among Japanese, Filipino, Chinese, NHOPI, and white populations in Hawai‘i.

**Methods:**

We studied 18,200 subjects aged 18 or older from the Hawai‘i Behavioral Risk Factor Surveillance System. We performed Poisson regression analyses to examine the prevalence of diabetes by race/ethnicity, age, sex, marital status, education, income, health care coverage, obesity, smoking and drinking status, physical activity, and fruit and vegetable consumption and examined the interactions of these factors with age and race/ethnicity.

**Results:**

We found disparities in diabetes prevalence among respondents aged 35 to 44 and among Asians and NHOPIs, and disparities increased with age. NHOPIs and Filipinos had the highest prevalence of diabetes after controlling for other demographic factors and lifestyle variables. Japanese adults were less likely than NHOPIs and Filipinos to have diabetes; however, whites had the lowest prevalence. Income, physical activity, and obesity were the strongest predictors of diabetes.

**Conclusion:**

NHOPIs and Filipinos have higher rates of diabetes compared with other races/ethnicities in Hawai‘i. More research is needed to reduce diabetes disparities among NHOPI and Filipino populations in Hawai‘i. This study also shows the importance of conducting age-specific analyses of racial/ethnic-subgroups for health disparities.

**Summary**

**What is already known about this topic?**

The prevalence of diabetes varies significantly among racial/ethnic groups in Hawai‘i. However, how prevalence varies by age for Asian subgroups and Native Hawaiian/Other Pacific Islanders (NHOPIs) is understudied.

**What is added by this report?**

We used the Hawai‘i Behavioral Risk Factor Surveillance System to examine diabetes prevalence by age and race/ethnicity and assessed how socioeconomic status and lifestyle behaviors affected prevalence among Japanese, Filipino, Chinese, NHOPI, and white populations in Hawai‘i.

**What are the implications for public health practice?**

NHOPIs and Filipinos have higher rates of diabetes compared with other races/ethnicities in Hawai‘i. This study shows the importance of conducting age-specific analyses of racial/ethnic-subgroups for health disparities.

## Introduction

Diabetes has reached epidemic proportions in the United States (1). It affects approximately 30 million Americans (9.4%), 21.3 million diagnosed and another 7.2 million undiagnosed (2). Diabetes is more prevalent among racial/ethnic minority populations, especially those of indigenous origin, who have higher rates of complications and other disorders from diabetes than do nonminority populations (3,4). In the United States in 2017, 10.3% of the Asian population had diabetes compared with 7.3% of the white population (2).

Hawai‘i is a multicultural state in which Asians, Native Hawaiians, and Other Pacific Islanders (NHOPIs) make up two-thirds of the population. In 2010, the state’s population was 1,360,301, and the estimated racial/ethnic distribution was 21.3% Native Hawaiian, 2.7% Other Pacific Islander, 22.7% white, 16.3% Japanese, 17.2% Filipino, and 6.8% Chinese (5). Diabetes is most prevalent among racial/ethnic minority populations in Hawai‘i. In 2014, an estimated 12.8% of Native Hawaiians, 10.0% of Chinese, 13.0% of Filipinos, 13.6% of Japanese, and 14.9% of Other Pacific Islanders were diagnosed with diabetes compared with 5.0% of white residents of the state (6). Previous research examined diabetes prevalence across Asian subpopulations; however, none examined interactions related to age or race/ethnicity (7–10). Such information is needed, because age distribution differs significantly across population groups, and diabetes risk increases with age. For example, although Native Hawaiians make up 21.3% of Hawai‘i’s population, they are only 10.9% of the population aged 60 or older. In contrast, Japanese make up 16.3% of the state’s population but 37.6% of residents aged 60 or older (5).

Other reasons for diabetes-related disparities among NHOPI and Asian subgroups in Hawai‘i are associated with biological, health care system, behavioral, socioeconomic, cultural, and environmental factors (8,10,11). For example, traditional NHOPI diets have shifted from locally sourced foods low in fat and high in fiber to processed foods that are high in fat, salt, calories, and sugar (11). NHOPIs have the lowest levels of educational attainment, lowest mean income, highest rates of poverty, and highest prevalence of being current, everyday smokers compared with white, Japanese, and Chinese adults in Hawai‘i (12). NHOPIs also have more difficulty accessing Westernized health care services because of socioeconomic disparities, cultural preferences, and discrimination (13). Yet, no studies have examined diabetes prevalence across race/ethnicity by age and the extent to which lifestyle behaviors affect diabetes among those who reside in Hawai‘i. Although eliminating diabetes disparities may not be possible (eg, because of genetic issues), such research may improve health disparities by enabling a better understanding of interactions related to age and race/ethnicity and the role of modifiable health behaviors.

By using 3 waves of population-representative data from the Hawai‘i Behavioral Risk Factor Surveillance System (HBRFSS) (14), we aimed to 1) describe racial/ethnic differences in diabetes prevalence by age groups among NHOPI, white, and 3 Asian subgroups (Japanese, Chinese, and Filipino) and 2) assess the relationship between associated risk factors and the prevalence ratios of diabetes. We hypothesized that age, race/ethnicity, socioeconomic status, and risky health behaviors are strongly associated with diabetes.

## Methods

### Data source

The Behavioral Risk Factor Surveillance System (BRFSS), is an annual telephone survey and a collaborative project between US states and the Centers for Disease Control and Prevention. BRFSS collects data on health risk behaviors, chronic diseases, and access to health care. HBRFSS started in 1986 with results reported annually. Participants are noninstitutionalized residents of Hawai‘i aged 18 or older. HBRFSS collects detailed racial/ethnic data, including a breakdown of Asian subgroups. Since 2011, participants are randomly selected from houses with listed and unlisted landline and cellular telephone numbers. HBRFSS uses the weighting methodology known as iterative proportional fitting or raking (15). Raking allows the introduction of more demographic variables into the statistical weighting process, and the resulting adjusted sample weights provide a closer match between the sample and the population. BRFSS provides valid national estimates, within-state estimates, and comparisons across states (16). This study was deemed exempt and approved by the University of Hawai‘i Institutional Review Board.

### Outcome variable — diabetes

To determine diabetes status, participants were asked if a doctor, nurse, or other health professional ever told them they had diabetes (yes/no). We described independent variables in 3 categories — demographics, obesity, and lifestyle factors.

Demographic variables were race/ethnicity, age, sex, marital status, education level, annual household income, and health care coverage. The Hawai‘i Department of Health provides detailed information on categorizing race/ethnicity of HBRFSS respondents (17). Because of the limited number of respondents classified as Other Pacific Islander, we combined that population with those classified as Native Hawaiian into an NHOPI group. We focused on the 5 largest racial/ethnic groups in Hawai‘i: 1) white, 2) NHOPI, 3) Filipino, 4) Japanese, and 5) Chinese. Participants were categorized into 7 age groups (18–24, 25–34, 35–44, 45–54, 55–64, 65–74, and ≥75 y). Sex was self-reported by the participant as male or female. Marital status was coded as married, divorced/separated, never married, or widowed. Educational attainment was based on the highest grade or year of school completed (eg, less than high school, high school/general equivalency diploma, 1–3 y of college, or ≥4 y of college). Health coverage was coded as yes (having any kind of health care coverage) or no. Income was based on the participant’s annual household income from all sources (≤$14,999, $15,000–$24,999, $25,000–$49,999, $50,000–$74,999, ≥$75,000, or unknown). The category of “unknown” was kept in the model because of missing data for income.

Participants were asked questions about height and weight to calculate body mass index (weight in kg divided by height in m^2^ [BMI]): About how much do you weigh without shoes?, About how tall are you without shoes?. Height and weight were used to estimate BMI, and participants whose BMI was greater than or equal to 30 kg/m^2^ were categorized as obese (18).

Lifestyle variables were smoking status, heavy drinking status, physical activity per week, and daily fruit and vegetable consumption. Smoking status was coded as never, smoke some days, smoke every day, and former smoker (ie, participants who smoked at least 100 cigarettes in their entire life, but no longer smoke at all). Participants were asked to provide the number of days per week or per month during the past 30 days that they had at least one alcoholic beverage and the average number of alcohol drinks per day. We used only the “heavy drinking” variable, defined as more than 2 drinks per day for men or more than 1 drink per day for women (yes/no).

Participants reported the amount of time they spent per week participating in physical activities (eg, walking, gardening, running) outside of work. The physical activity variable was coded on the basis of US physical activity guidelines as nothing, less than guidelines (1–149 min/wk), meets guidelines (150–300 min/wk), and exceeds guidelines (>300 min/wk) (19). Participants were asked the number of times per day, week, or month they ate fruit (fresh, canned, frozen) and vegetables (dark green or orange-colored). Fruit and vegetable consumption variables were coded as none, 1 to 2 times per day, or 3 or more times per day.

### Statistical analysis

HBRFSS reported physical activity and daily fruit and vegetable consumption only for odd years, therefore we used 2011, 2013, and 2015 data for our study. HBRFSS responses were 7,606 (44.8%) for 2011, 7,858 (40.2%) for 2013, and 7,163 (42.2%) for 2015 (14) for a total of 22,627. From these, we excluded those missing values for race/ethnicity (n = 1,966); diabetes (n = 36); age, sex, marital status, and health coverage (n = 261); smoking and drinking (n = 885); obesity (n = 469); and lifestyle variables (n = 810). The 3 years of survey data yielded a total sample size of 18,200. Sensitivity analysis was performed by including the missing values as separate “missing” categories, and results remained similar.

Sample characteristics by race/ethnicity were analyzed by accounting for complex survey weights and design strata. We conducted univariate analysis of the whole sample and bivariate analysis of frequency and weighted prevalence of diabetes by all independent variables. We performed weighted Poisson regression analyses of the crude model and 3 multivariate prevalence ratios (PRs) and the corresponding 95% confidence intervals (CIs). We estimated the PRs (direct estimate of the ratio between 2 groups) instead of odds ratios (ORs), because ORs tend to have a larger effect size when the outcome event is common (20–22).

We used 3 multivariate main effect models. Model 1 adjusted for demographic and socioeconomic variables, and model 2 added obesity and lifestyles. Because of potential interaction effects between age and other demographic variables (especially education and income), interactions effects were checked, and deviance testing showed a significant interaction effect for age and race/ethnicity (*P* < .001). Thus, Model 3 examined the age and race/ethnicity interaction effect while adjusting for all other variables. Statistical software R, version 3.4.1 (The R Foundation) and its libraries “survey,” “effects,” and “ggplot2” were used for the analyses. Significance was set at *P* < .05.

## Results

NHOPIs had the highest weighted proportion of adults aged 18 to 34 but the lowest weighted proportion aged 75 or older (Table 1). They also had the lowest proportions of adults reporting a college education and an annual household income at or greater than $75,000. Japanese and Chinese participants had the lowest weighted proportions of adults aged 18 to 34 and the highest proportions aged 75 and older, and these groups also had the highest proportions of adults reporting any college education and an annual household income at or greater than $75,000.

**Table 1:** Demographic Characteristics of Participants (N = 18,200) and Lifestyle Risk Factors for Diabetes, by Race/Ethnicity, Hawai‘i Behavioral Risk Factor Surveillance System, 2011, 2013, 2015^a^

Variable	White	Native Hawaiian/Other Pacific Islander	Filipino	Japanese	Chinese
**Age, y**					
18–24	306 (9.4)	335 (17.9)	217 (13.4)	161 (7.6)	67 (14.3)
25–34	685 (18.0)	503 (25.2)	310 (19.2)	258 (9.3)	85 (12.3)
35–44	801 (16.0)	485 (20.0)	363 (20.0)	314 (11.5)	117 (15.6)
45–54	1,254 (18.4)	487 (13.1)	411 (16.3)	594 (17.0)	166 (17.3)
55–64	2,156 (17.7)	486 (11.8)	435 (15.0)	960 (21.8)	219 (16.8)
65–74	1,823 (12.5)	373 (7.7)	351 (9.7)	813 (14.8)	179 (11.6)
≥75	1,024 (8.0)	184 (4.4)	209 (6.4)	885 (18.1)	184 (12.1)
**Sex**					
Female	4,156 (45.6)	1,639 (50.9)	1,303 (52.2)	2,198 (52.3)	551 (49.0)
Male	3,893 (54.4)	1,214 (49.1)	993 (47.8)	1,787 (47.7)	466 (51.0)
**Marital status**					
Married	4,436 (59.1)	1,388 (46.7)	1,310 (57.3)	2,117 (54.3)	564 (55.8)
Divorced/separated	1,520 (13.5)	403 (10.6)	209 (7.5)	453 (10.0)	124 (8.0)
Never married	1,290 (21.7)	798 (36.7)	530 (28.3)	863 (25.2)	214 (28.3)
Widowed	803 (5.70	264 (5.9)	247 (6.8)	552 (10.5)	115 (7.9)
**Education level**					
<High school	239 (7.0)	197 (16.1)	203 (15.0)	76 (3.8)	16 (3.20)
High school diploma or GED	1,648 (25.2)	1,300 (44.2)	805 (33.5)	929 (25.5)	178 (20.6)
College, 1–3 years	2,250 (34.2)	813 (28.5)	621 (33.6)	1,105 (35.9)	248 (32.3)
College, ≥4 years	3,912 (33.5)	543 (11.1)	667 (18.0)	1,875 (34.7)	575 (43.9)
**Annual income, $**					
≤14,999	711 (7.0)	427 (14.4	269 (8.5)	183 (3.6)	65 (6.0)
15,000–24,999	991 (11.7)	547 (19.7	394 (16.1)	411 (8.8)	80 (6.4)
25,000–49,999	1,774 (20.6)	754 (25.0	719 (30.8)	956 (22.3)	221 (21.8)
50,000–74,999	1,306 (16.4)	383 (13.3		706 (17.0)	181 (16.1)
≥75,000	2,747 (37.4)	533 (18.8	431 (20.7)	1,362 (39.1)	373 (38.5)
Unknown	520 (6.9)	209 (8.8	186 (10.5)	367 (9.2)	97 (11.2)
**Health care coverage**					
Yes	7,506 (92.7)	2,558 (86.8	2,096 (90.6)	3,843 (95.9)	960 (93.7)
No	543 (7.3)	295 (13.2	200 (9.4)	142 (4.1)	57 (6.3)
**Obese^b^**					
Yes	1,540 (20.6)	1,228 (43.5	426 (18.8)	598 (16.6)	110 (9.9)
No	6,509 (79.4)	1,625 (56.5	1,870 (81.2)	3,387 (83.4)	907 (90.1)
**Smoking status^c^**					
Never smoker	4,176 (54.1)	1,534 (53.3	1,529 (66.5)	2,390 (60.1)	744 (75.8)
Former smoker	2,875 (31.8)	737 (23.0	492 (21.0)	1,215 (28.5)	216 (17.4)
Smoke some days	293 (4.5)	172 (6.9	96 (4.6)	87 (2.8)	10 (1.0)
Smoke every day	705 (9.6)	410 (16.9	179 (7.9)	293 (8.5)	47 (5.8)
**Heavy drinking^d^**					
Yes	807 (9.8)	283 (10.9	108 (5.7)	184 (5.1)	36 (3.2)
No	7,242 (90.2)	2,570 (89.1	2,188 (94.3)	3,801 (94.9)	981 (96.8)
Don’t drink	1,373 (16.7)	706 (25.0	615 (27.5)	888 (23.5)	194 (21.3)
**Meets US guidelines for physical activity^e^**					
No physical activity	1,373 (16.7)	706 (25)	615 (27.5)	888 (23.5)	194 (21.3)
Less than guidelines	1,203 (17.6)	473 (16.1	475 (22.6)	775 (22.6)	226 (23.6)
Meets guidelines	1,610 (21.7)	521 (17.0	405 (18.7)	749 (19.1)	206 (19.0)
Exceeds guidelines	3,863 (44.0)	1,153 (42.0	801 (31.2)	1,573 (34.9)	391 (36.1)
**Daily servings of fruit**					
None	2,394 (33.3)	1,276 (46.6	918 (42.8)	1,623 (44.9)	340 (39.5)
1–2	4,344 (52.3)	1,156 (38.5	1,024 (42.8)	2,006 (47.6)	551 (50.4)
≥3	1,311 (14.3)	421 (14.9	354 (14.4)	356 (7.5)	126 (10.2)
**Daily servings of vegetables**					
None	1,133 (17.1)	742 (27.4	608 (29.5)	839 (22.6)	196 (20.5)
1–2	4,999 (61.0)	1,541 (52.1	1,217 (51.6)	2,385 (61.0)	627 (62.4)
≥3	1,917 (21.9)	570 (20.5	471 (18.9)	761 (16.4)	194 (17.1)

Abbreviation: GED, general equivalency diploma.

a Values are number (weighted percentage). All *P* values are < .001 and were calculated by χ^2^ test.

b Body mass index (weight in kg divided by height in m^2^) ≥30.

c Participants who smoked at least 100 cigarettes in their entire life, but no longer smoke at all.

d Defined as more than 2 drinks per day for men or more than 1 drink per day for women.

e Less than guidelines = 1–149 min/wk, meets guidelines = 150–300 min/wk), and exceeds guidelines = >300 min/wk (19).

Diabetes prevalence was 11.5% (95% CI, 10.2%–12.9%) for Japanese, 11.2% (95% CI, 9.5%–13.2%) for Filipinos, 9.9% (95% CI, 8.6%–11.3%) for NHOPIs, 9.1% (95% CI, 6.7%–12.1%) for Chinese, and 5.4% (95% CI, 4.8%–6.1%) for whites (Table 2). The weighted prevalence of diabetes was highest among adults aged 65 to 74 (18.6%; 95% CI, 16.6%–20.7%) and 75 or older (17.6%; 95% CI, 15.3%–20.0%), who were widowed (18.4%; 95% CI, 15.6%–21.4%), had less than a high school diploma or general equivalency diploma (13.1%; 95% CI, 10.1%–16.6%), had an annual household income of less than $15,000 (12.6%; 95% CI, 10.3%–15.3%), had health care coverage (9.1%; 95% CI, 8.5%–9.7%), were obese (16.5%; 95% CI, 15.0%–18.1%), were former smokers (11.8%; 95% CI, 10.6%–13.0%), participated in no physical activity (13.2%; 95% CI, 11.7%–14.8%), and consumed no vegetables (10.4%; 95% CI, 9.1%–11.8%).

**Table 2:** Prevalence Ratios, by Demographic Characteristics and Lifestyle Risk Factors of All Participants (N = 18,200) and Participants With Diabetes (N = 1,882), Hawai‘i Behavioral Risk Factor Surveillance System, 2011, 2013, 2015^a^

Variable	All Participants, N (%)	Participants With Diabetes, N (%) [95% Confidence Interval]	*P* Value
**Race/ethnicity**			
White	8,049 (37.1)	570 (5.4) [4.8–6.1]	<.001
Native Hawaiian/Other Pacific Islander	2,469 (12.5)	326 (9.9) [8.6–11.3]	
Filipino	2,296 (17.4)	321 (11.2) [9.5–13.2]	
Japanese	3,985 (23.5)	519 (11.5) [10.2–12.9]	
Chinese	1,017 (6.8)	100 (9.1) [6.7–12.1]	
**Age, y**			
18–24	1,086 (11.3)	6 (0.5) [0.2–1.2]	<.001
25–34	1,841 (16.9)	33 (1.7) [1.0–2.5]	
35–44	2,080 (16.2)	110 (5.0) [3.8–6.3]	
45–54	2,912 (16.8)	246 (8.4) [7.0–10.1]	
55–64	4,256 (17.2)	496 (13.5) [11.9–15.1]	
65–74	3,539 (11.8)	576 (18.6) [16.6–20.7]	
≥75	2,486 (9.8)	415 (17.6) [15.3–20.0]	
**Sex**			
Female	9,847 (49.4)	996 (8.9) [8.1–9.8]	.60
Male	8,353 (50.6)	886 (8.6) [7.9–9.5]	
**Marital status**			
Married	9,815 (55.5)	1,004 (9.3) [8.5–10.1]	<.001
Divorced/separated	2,709 (10.8)	282 (9.9) [8.2–11.9]	
Never married	3,695 (26.4)	243 (4.7) [3.9–5.6]	
Widowed	1,981 (7.2)	353 (18.4) [15.6–21.4]	
**Education level**			
<High school	731 (8.8)	123 (13.1) [10.1–16.6]	<.001
High school diploma or GED	4,860 (29.3)	573 (9.1) [8.2–10.2]	
College, 1–3 y	5,037 (33.5)	532 (9.1) [8.1–10.2]	
College, ≥4 y	7,572 (28.4)	654 (6.7) [6.1–7.4]	
**Annual household income, $**			
≤14,999	1,655 (7.5)	242 (12.6) [10.3–15.3]	<.001
15,000–24,999	2,423 (12.6)	304 (9.8) [8.2, 11.7]	
25,000–49,999	4,424 (23.5)	496 (9.9) [8.7–11.2]	
50,000–74,999	2,873 (15.5)	259 (7.9) [6.5–9.4]	
≥75,000	5,446 (32.1)	429 (6.8) [6.0–7.7]	
Unknown	1,379 (8.6)	152 (9.9) [7.7–12.5]	
**Health care coverage**			
Yes	16,963 (92.3)	1,802 (9.1) [8.5–9.7]	.001
No	1,237 (7.7)	80 (5.5) [3.9–7.4]	
**Obese^b^**			
Yes	3,902 (22.1)	770 (16.5) [15.0–18.1]	<.001
No	14,298 (77.9)	1,112 (6.6) [6.0–7.2]	
**Smoking status^c^**			
Never smoker	10,373 (59.0)	956 (7.9) [7.2–8.6]	<.001
Former smoker	5,535 (26.8)	711 (11.8) [10.6–13.0]	
Smoke some days	658 (4.2)	53 (5.9) [3.8–8.7]	
Smoke every day	1,634 (9.9)	162 (7.4) [5.8–9.2]	
**Heavy drinking^d^**			
Yes	1,418 (7.7)	86 (5.0) [3.7–6.6]	<.001
No	16,782 (92.3)	1,796(9.1) [8.5–9.7]	
**Meets US guidelines for physical activity^e^**			
No physical activity	3,776 (21.7)	568 (13.2) [11.7–14.8]	<.001
Less than guidelines	3,152 (19.8)	305 (7.7) [6.5–9.0]	
Meets guidelines	3,491 (19.7)	307 (7.3) [6.1–8.5]	
Exceeds guidelines	7,781 (38.8)	702 (7.7) [6.9–8.5]	
**Daily servings of fruit**			
None	6,551 (40.1)	724 (9.1) [8.2–10.1]	.11
1–2	9,081 (47.3)	910 (8.2) [7.4–9.0]	
≥3	2,568 (12.6)	248 (10.0) [8.2–12.0]	
**Daily servings of vegetables**			
None	3,518 (22.4)	462 (10.4) [9.1–11.8]	.001
1–2	10,769 (58.1)	1,048 (8.4) [7.7–9.2]	
≥3	3,913 (19.5)	372 (8.1) [7.0–9.3]	

Abbreviation: GED, general equivalency diploma.

a Percentages are weighted.

b Body mass index (weight in kg divided by height in m^2^) ≥30.

c Participants who smoked at least 100 cigarettes in their entire life, but no longer smoke at all.

d Defined as more than 2 drinks per day for men or more than 1 drink per day for women.

e Less than guidelines = 1–149 min/wk, meets guidelines = 150–300 min/wk, and exceeds guidelines = >300 min/wk (19).

In the crude model all variables were associated with diabetes prevalence except for sex, current smoking status, and daily fruit consumption (Table 3). Compared with whites, Japanese (PR = 2.12; 95% CI, 1.81–2.49) and Filipino (PR = 2.07; 95% CI, 1.69–2.52) had the highest crude PRs of diabetes. When all demographic variables were adjusted for, the PR for Japanese reduced to 1.69 (95% CI, 1.44–1.99) (model 1) and 1.77 (95% CI, 1.51–2.08) when all variables were adjusted for (model 2). The PR for NHOPI changed from 1.82 (95% CI, 1.52–2.17) in the crude model to 2.23 (95% CI, 1.87–2.66) in model 1 and 1.74 (95% CI, 1.46–2.08) in model 2. Marital status, education, and health insurance were no longer significant after adjusting for other demographics (model 1). After adjusting for all variables (model 2), race/ethnicity, age, household income, and obesity were strongly associated with diabetes. Physical activity and heavy drinking were significant for protective factors.

**Table 3:** Crude and Multivariate Prevalence Ratios of Diabetes (N = 18,200), Hawai’i Behavioral Risk Factor Surveillance System, 2011, 2013, 2015

Variable	Crude Model		Multivariate Model 1^a^		Multivariate Model 2^b^		Multivariate Model 3^c^	
	Prevalence Ratio (95% Confidence Interval)	*P* Value	Prevalence Ratio (95% Confidence Interval)	*P* Value	Prevalence Ratio (95% Confidence Interval)	*P* Value	Prevalence Ratio (95% Confidence Interval)	*P* Value
**Race/ethnicity **								
White	1 [Reference]							
Native Hawaiian/Other Pacific Islander	1.82 (1.52–2.17)	<.001	2.23 (1.87–2.66)	<.001	1.74 (1.46–2.08)	<.001	—c	—c
Filipino	2.07 (1.69–2.52)	<.001	2.12 (1.75–2.56)	<.001	2.16 (1.79–2.61)	<.001	—c	—c
Japanese	2.12 (1.81–2.49)	<.001	1.69 (1.44–1.99)	<.001	1.77 (1.51–2.08)	<.001	—c	—c
Chinese	1.68 (1.24–2.28)	<.001	1.69 (1.26–2.26)	<.001	1.90 (1.43–2.53)	<.001	—c	—c
**Age, y**								
18–24	0.10 (0.04–0.27)	<.001	0.08 (0.03–0.21)	<.001	0.10 (0.04–0.27)	<.001	—c	—c
25–34	0.34 (0.21–0.54)	<.001	0.31 (0.19–0.50)	<.001	0.34 (0.21–0.55)	<.001	—c	—c
35–44	1 [Reference]							
45–54	1.70 (1.27–2.29)	.004	1.82 (1.36–2.43)	<.001	1.85 (1.39–2.47)	<.001	—c	—c
55–64	2.72 (2.09–3.54)	<.001	2.88 (2.21–3.75)	<.001	3.04 (2.34–3.94)	<.001	—c	—c
65–74	3.75 (2.89–4.87)	<.001	3.91 (3.01–5.09)	<.001	4.14 (3.19–5.38)	<.001	—c	—c
≥75	3.56 (2.71–4.66)	<.001	3.42 (2.57–4.56)	<.001	4.04 (3.02–5.41)	<.001	—c	—c
**Sex**								
Female	1 [Reference]							
Male	0.97 (0.85–1.10)	.60	1.17 (1.03–1.32)	.01	1.12 (0.99–1.27)	.08	1.13 (0.99– 1.28)	.06
**Marital status**								
Married	1 [Reference]							
Divorced/separated	1.07 (0.87–1.31)	.51	0.91 (0.74–1.12)	.36	0.89 (0.74–1.09)	.26	0.89 (0.73– 1.08)	.25
Never married	0.50 (0.41–0.61)	<.001	1.11 (0.91–1.35)	.29	1.07 (0.88–1.29)	.49	1.06 (0.88– 1.28)	.56
Widowed	1.98 (1.66–2.36)	<.001	1.00 (0.83–1.20)	.997	1.00 (0.83–1.21)	.98	1.01 (0.84– 1.21)	.94
**Education level**								
<High school	1 [Reference]							
High school diploma or GED	0.70 (0.53–0.91)	.008	0.94 (0.73–1.21)	.61	0.99 (0.77–1.29)	.96	1.02 (0.78– 1.32)	.90
College, 1–3 years	0.70 (0.53–0.91)	.008	0.95 (0.73–1.23)	.70	1.02 (0.78–1.33)	.87	1.04 (0.79– 1.36)	.77
College, ≥4 years	0.51 (0.40–0.67)	<.001	0.75 (0.57–0.98)	.03	0.88 (0.67–1.16)	.37	0.88 (0.67– 1.16)	.38
**Annual household income, $**								
≤14,999	1 [Reference]							
15,000–24,999	0.78 (0.60–1.01)	.055	0.73 (0.57–0.94)	.01	0.72 (0.57–0.91)	.006	0.72 (0.57– 0.91)	.001
25,000–49,999	0.78 (0.62–0.98)	.04	0.66 (0.53–0.82)	..002	0.63 (0.51–0.79)	<.001	0.64 (0.51– 0.79)	<.001
50,000–74,999	0.62 (0.48–0.81)	.004	0.58 (0.45–0.75)	<.001	0.55 (0.43–0.71)	<.001	0.55 (0.43– 0.71)	<.001
≥75,000	0.54 (0.43–0.68)	<.001	0.54 (0.43–0.68)	<.001	0.53 (0.42–0.67)	<.001	0.53 (0.42– 0.67)	<.001
Unknown	0.78 (0.58–1.06)	.11	0.77 (0.58–1.01)	.06	0.74 (0.56–0.97)	.03	0.74 (0.56– 0.97)	
**Health care coverage**								
Yes	1 [Reference]							
No	0.61 (0.44–0.83)	.002	0.84 (0.62–1.14)	.26	0.91 (0.67–1.24)	.56	0.92 (0.68– 1.26)	.62
**Obese^d^**								
No	1 [Reference]							
Yes	2.51 (2.20–2.85)	<.001	—e	—e	2.55 (2.24–2.90)	<.001	2.53 (2.22– 2.89)	<.001
**Smoking status^f^**								
Never smoker	1 [Reference]							
Former smoker	1.50 (1.31–1.72)	<.001	—e	—e	1.10 (0.96–1.25)	.16	1.10 (0.96– 1.25)	.17
Smoke some days	0.76 (0.51–1.13)	.17	—e	—e	1.02 (0.72–1.46)	.89	1.02 (0.72– 1.45)	.92
Smoke every day	0.94 (0.74–1.20)	.61	—e	—e	0.97 (0.77–1.24)	.83	0.97 (0.76– 1.23)	.81
**Heavy drinking^g^**								
No	1 [Reference]							
Yes	0.55 (0.41–0.73)	<.001	—e	—e	0.75 (0.56–1.00)	.05	0.74 (0.55– 0.98)	.04
**Physical activity, US guidelines^h^**								
No physical activity	1 [Reference]							
Less than US. guidelines	0.58 (0.48–0.71)	<.001	—e	—e	0.82 (0.68–0.99)	.04	0.81 (0.67– 0.97)	.03
Meets guidelines	0.55 (0.45–0.67)	<.001	—e	—e	0.79 (0.65–0.95)	.02	0.79 (0.65– 0.96)	.02
Exceeds guidelines	0.58 (0.50–0.68)	<.001	—e	—e	0.71 (0.61–0.83)	<.001	0.71 (0.61– 0.83)	<.01
**Daily servings fruit**								
None	1 [Reference]							
1-2	0.90 (0.78–1.03)	.13	—e	—e	0.92 (0.80–1.06)	.25	0.92 (0.80– 1.06)	.27
≥3	1.09 (0.89–1.35)	.41	—e	—e	1.17 (0.95–1.44)	.13	1.17 (0.95– 1.44)	.13
**Daily servings vegetables**								
None	1 [Reference]							
1–2	0.81 (0.69–0.95)	.009	—e	—e	0.95 (0.81–1.11)	.49	0.95 (0.81– 1.11)	.49
≥3	0.78 (0.64–0.94)	.01	—e	—e	0.93 (0.76–1.14)	.49	0.94 (0.77– 1.15)	.55

Abbreviation: GED, general equivalency diploma.

a Weighted multivariate Poisson model adjusted for demographic and socioeconomic variables.

b Weighted multivariate Poisson model adjusted for all independent variables (including obesity and lifestyle variables).

c Weighted multivariate Poisson model with age and race/ethnicity interaction effects adjusted for all independent variables. Results for race/ethnicity and age are available from the authors.

d Body mass index (weight in kg divided by height in m^2^) ≥30.

e Cells are blank because multivariate model 1 included only demographic variables.

f Participants who smoked at least 100 cigarettes in their entire life, but no longer smoke at all.

g Defined as more than 2 drinks per day for men or more than 1 drink per day for women.

h Less than guidelines = 1–149 min/wk, meets guidelines = 150–300 min/wk, and exceeds guidelines = >300 min/wk (19).

To illustrate variability by age and race/ethnicity, we graphically presented diabetes prevalence and 95% CIs (Figure) from model 3, results of which are available from the authors. Differences in diabetes prevalence appeared by age 35. For instance, whites had significantly lower diabetes prevalence from age 35 or older than Filipinos and NHOPIs (*P* < .05). Compared with Japanese participants, whites had significantly lower diabetes prevalence for ages 35 to 74 (*P* < .05). NHOPIs and Filipinos had higher diabetes prevalence than Japanese participants. For example, NHOPIs had a significantly higher diabetes prevalence at ages 45 to 54 (*P* = .01). However, NHOPIs aged 55 to 64 had higher prevalence in diabetes than Japanese participants (*P* = .06). Additionally, Filipinos aged 55 and older had significantly higher diabetes prevalence than Japanese participants (55–64 y, *P* = .03; 65–74 y, *P* = .008; ≥75, *P* = .006).

**Figure:**
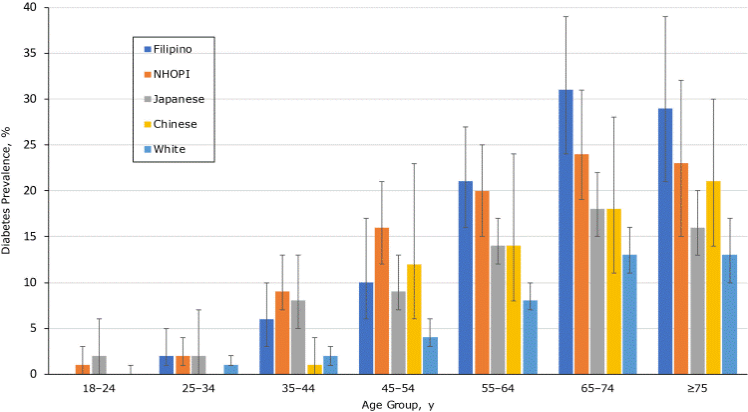
Interaction between age and race/ethnicity in diabetes prevalence among Native Hawaiian/Other Pacific Islanders and Asian subpopulations (N = 18,200), calculated as crude and multivariate prevalence ratios. Estimates are from weighted Poisson regression analyses (age–race/ethnicity interaction effect, *P* < .001). Nonoverlapping CIs indicate significant differences at 5. Source: Hawai‘i Behavioral Risk Factor Surveillance System, 2011, 2013, 2015 (14). Abbreviations: NHOPI, Native Hawaiian and Other Pacific Islander.

**Table:** 

Race/Ethnicity	Age, y, Prevalence Ratio (95% Confidence Interval)						
	18–24	25–34	35–44	45–54	55–64	65–74	≥75
Filipino	0	0.02 (0.01–0.05)	0.06 (0.03–0.10)	0.10 (0.06–0.17)	0.21 (0.16–0.27)	0.31 (0.24–0.39)	0.29 (0.21–0.39)
NHOPI	0.01 (0.00–0.03)	0.02 (0.01–0.04)	0.09 (0.07–0.13)	0.16 (0.12–0.21)	0.20 (0.15–0.25)	0.24 (0.19–0.31)	0.23 (0.15–0.32)
Japanese	0.02 (0.00–0.06)	0.02 (0.00–0.07)	0.08 (0.05–0.13)	0.09 (0.07–0.13)	0.14 (0.12–0.17)	0.18 (0.15–0.22)	0.16 (0.13–0.20)
Chinese	0	0	0.01 (0.00–0.04)	0.12 (0.06–0.23)	0.14 (0.08–0.24)	0.18 (0.11–0.28)	0.21 (0.14–0.30)
White	0 (0–0.01)	0.01 (0.01–0.02)	0.02 (0.01–0.03)	0.04 (0.03–0.06)	0.08 (0.07–0.10)	0.13 (0.11–0.16)	0.13 (0.10–0.17)

We saw minimal changes in PRs between models 2 and 3 for all other independent variables. We calculated PRs and 95% CIs of diabetes by risk factors calculated from model 3 (Table 3). The PRs of diabetes decreased as household income increased. For example, the PR of diabetes among participants whose annual household income was greater than or equal to $75,000 was 0.53 (95% CI, 0.42–0.67) compared with those with an annual household income of less than $15,000. Participants who were obese had the highest diabetes PR at 2.53 (95% CI, 2.22–2.89) compared with those who were not obese. Participants who drank heavily had a lower PR of 0.74 (95% CI, 0.55–0.98) compared with those who did not. Participants who participated in more physical activity also had a lower diabetes PR, and those who exceeded the US physical activity guidelines were 0.71 (95% CI, 0.61–0.83) times as likely to have diabetes compared with those who participated in no physical activity.

## Discussion

We found that NHOPI, Filipino, Japanese, and Chinese residents of Hawai‘i all have significantly higher diabetes PRs than white residents. These disparities remain after adjusting for demographics and diabetes risk factors. Furthermore, we found that the association between age and diabetes varied by race/ethnicity, with diabetes prevalence increasing more rapidly with age among NHOPI, Filipino, and Japanese residents than among white residents. To our knowledge, this is the first study to examine relationships between age and race/ethnicity for diabetes among NHOPI and Asian subpopulations. Our results illustrate the need for researchers to disaggregate and further define terms like “ethnic” and “minority” in discussing populations. Our findings also highlight the large burden of diabetes and its associated risk factors among NHOPI and Asian residents of Hawai‘i.

Our findings affirm the importance of including age in analyses by race/ethnicity, because different racial/ethnic groups may have different age distributions. When only race/ethnicity was considered, Filipinos had the greatest PRs of diabetes after adjusting for demographics (model 1) and all risk factors (model 2). However, when age was included in the analyses by race/ethnicity, variations in diabetes prevalence were found. NHOPI and Filipino residents had significantly higher diabetes prevalence starting at age 35 than white residents of the state. Furthermore, NHOPIs and Filipinos had significantly higher diabetes prevalence than Japanese residents in specific age groups.

Our results are consistent with predominant findings that higher diabetes risk is associated with low household income, obesity, and lack of physical activity (23,24). Adults with greater household income who were not obese and who exceeded US physical activity guidelines were less likely to have diabetes. Although marital status, educational attainment, health coverage, former smoking, and vegetable consumption were significantly associated with diabetes prevalence in the bivariate analysis, these associations were no longer significant after adjusting for other demographics (model 1) and risk factors (model 2).

Strengths of this study are its focus on Hawai‘i, which allowed for obtaining a robust sample of NHOPI participants and Asian subgroups and for the examination of diabetes and other risk factors across Asian subgroups. In addition, this study focused on the differences in diabetes prevalence by age and race/ethnicity.

The study has limitations. First, BRFSS data are self-reported, and participants may not report accurate measures (ie, data can be over represented or underrepresented). Second, collapsing Native Hawaiians and Other Pacific Islanders into the one category limited our ability to compare them with separate Pacific Islander races/ethnicities, such as Samoans or Tongans. Third, categorizing each adult into a single ethnic group is problematic, because Hawai‘i has the highest proportion of multiracial residents in the United States (25). Fourth, this study did not have enough power to detect differences that may exist between racial/ethnic groups. The HBRFSS collects data on multiple races/ethnicities, and this should be a topic for future research. Lastly, we were unable to infer causation in the relationships between risk factors and diabetes because this was a cross-sectional study.

Future research is needed on how race/ethnicity is defined in health disparities research. Racial/ethnic-specific data collection and analyses are needed to investigate health disparities among heterogeneous groups that are often combined into one racial group (ie, Asian). Furthermore, demographic and risk factor variables did not account for all racial/ethnic disparities in diabetes prevalence. Future examination is needed of the role of genetic factors and body fat distribution in explaining high diabetes prevalence among NHOPI and Filipino populations in Hawai‘i (26). Public health programs are also needed to promote positive lifestyle behaviors before the high prevalence of obesity further increases diabetes rates among at-risk racial/ethnic populations. Public health programs in Hawai‘i should target education and early interventions, especially for Filipino and NHOPI residents by age 18 to 24, before diabetes disparities begin to appear.
